# French Academy of Science

**Published:** 1846-04

**Authors:** 


### FRENCH ACADEMY OF SCIENCE.

*Diseases of workmen employed in the fabrication of Lucifer Matches.* By T. Rousselle, M. D.—Dr. Rousselle called the attention of the authorities and of the public to the numerous diseases generated by the fabrication of lucifer matches. Dr. Gendrin has of late described a severe variety of bronchitis, prevalent amongst the workmen attached to the branch of industry referred to; and necrosis of the maxilla has also been looked upon by some German writers as a result of the noxious emanations in the wards of the factories. Dr. Rousselle remarked that, in the establishments in which all the operations relative to the fabrication of matches are performed in one room, cough and bronchitis affect the men indiscriminately, whatever may be their individual employment. But in those manufactories, in which the various branches of fabrication are carried on in separate rooms, those workmen only suffer who are exposed by the nature of their occupations to phosphoric vapours; and the intensity of the morbid effects produced appears to bear some ratio to the humidity of the weather and the degree of ventilation of the wards. As to the disease of the maxillary bones, two conditions have appeared to Dr. Rousselle to be indispensable to its production:—1. A sojourn in the factory of not less than two years; 2. The presence of decayed teeth. These special accidents are to be attributed, according to Dr. Rousselle, to the presence of phosporic acid, and perhaps of phosphorus in a gaseous condition in the atmosphere of the wards. He recommends, therefore, in order to prevent the production of the symptoms, a particular mode of ventilation, and the separation in distinct rooms of the various branches of fabrication.

*Hairy productions on the surface of the Tongue.* By Dr. Landousy, Professor of the Faculty of Rheims (see *Medical Times*, vol. xiii, p. 148.)—We mentioned in our communication, of November 15th, two cases of hairy productions of the tongue, brought before the Academy of Medicine by Dr. Landousy. One was a case of pleurisy; the other of erythema nodosum. Dr. Landousy has since met with fourteen similar instances: Cold abscess, 1; consumption, 3; pulmonary emphysema, 1; typhoid fever, 4; Bright's kidney, 1; icterus and cirrhosis, 1; uterine disease, 2; fracture, 1. In these sixteen cases, the black colour observed on the tongue was due to hairy productions which the naked eye could not distinguish from common hairs, but which presented under the microscope special characters. These hairs varied in length from one to fifteen millimetres, and in diameter from 1-5th to 1-200th part of a millimetre. The length of these hairs sometimes caused an unpleasant sensation in the throat, when they were vertically planted in the tongue, towards the apex of which they lie, on the contrary, in a horizontal direction.

*The Electric Maid.*—M. Arago also mentioned that he had been called upon to witness some of the most singular phenomena which he had ever beheld, in the shape of electric discharges of the most violent character, proceeding from the person of a young girl, aged thirteen, lately submitted to his inspection. This truly remarkable child overturns tables and chairs by merely touching them with her apron. When she sits down, the moment her feet touch the ground the chair is upset, and she is suddenly propelled with considerable force. M. Arago said he had seen all these experiments, and had not been able to detect any trick. He begged the Academy would appoint a committee to investigate the matter.—*Lon. Medical Times.*

